# Parents’ Attitudes and Adherence to Unintentional Injury Prevention Measures in Ankara, Turkey

**DOI:** 10.4274/balkanmedj.2016.1776

**Published:** 2017-08-04

**Authors:** Tolga İnce, Songül Yalçın, Kadriye Yurdakök

**Affiliations:** 1 Department of Pediatrics, Social Pediatrics Unit, Hacettepe University School of Medicine, Ankara, Turkey

**Keywords:** children, injury prevention, parents, unintentional injury

## Abstract

**Background:**

Childhood unintentional injuries are perceived as a leading public health issue since they are one of the preventable causes of paediatric mortality and morbidity. Whether and how parental factors are related to childhood injury has been researched insufficiently.

**Aims:**

To investigate parents’ attitudes to preventive measures of unintentional childhood injury, and the parental adherence to these measures.

**Study Design:**

Cross-sectional, descriptive study.

**Methods:**

The data were collected from the parents of children younger than ten years of age admitted to university hospital outpatient clinics for any reason and who agreed to be involved in the study. The first part of the questionnaire included sociodemographic profiles of participating children. Serious injuries were considered to be any injury that requires hospital admission. The second part of the questionnaire was prepared to evaluate parents’ adherence to injury prevention rules. A total score calculation about the adherence of the parents to the injury prevention rules was worked out the addition of the scores of each answer given in each age group. Answers for each item given by the parents were scored as wrong (0), sometimes (1) or correct (2). The score for each item was added and the result normalized to 100 points. Only complete questionnaires were used for analysis.

**Results:**

A total of 1126 children and parent pairs agreed to participate in the survey. It was found that 13.8% of the participating children had experienced at least one serious injury. Although three-quarters of the parents had received information about injury prevention, the overall injury prevention scores were found to be low. As children’s age increased, the total injury prevention scores of parents decreased significantly. Injury prevention scores were shown to increase significantly with high education and maternal occupation. However, scores were shown to decrease significantly with increased child age and family size.

**Conclusion:**

Our study shows that parental adherence to the child safety measures aimed at decreasing the unintentional injury risk of children is not satisfactory in Turkey. In particular, parents of 5-9-year-old children, big families (more than five people), parents with less than 8 years of education and non-working mothers should be the main target groups for intervention strategies according to our study results.

Childhood unintentional injuries are perceived as a leading public health issue because they are one of the preventable causes of paediatric mortality and morbidity. It is estimated that every day about 30.000 children are admitted to emergency departments due to injuries in the European Union (1,2). On the other hand, most childhood injuries could be prevented according to studies (3). Injury prevention programmes are specifically crucial for the improvement of child health and survival globally. The development of successful injury prevention programmes is related to the identification of risk factors for childhood injuries. Unfortunately, few countries have comprehensive data on unintentional childhood injuries.

Although it is known that behavioural, environmental, and social factors are important for injury prevention, whether and how parental factors related to childhood injury are searched insufficiently. So, understanding what parents do to prevent injuries, how well these prevention strategies work, and what encourages these actions is essential in order to decrease unintentional injury risk and to develop effective injury prevention strategies (4).

As in other developing countries, unintentional injuries are the fourth leading cause of death among children under five years of age, and they account for 1.5% of all paediatric deaths in Turkey (5,6). Recently it was found that the majority of unintentional injuries happen in the home in Turkey (7). Maternal educational background, mother’s occupation, the age of the child, the type of family, and monthly household income were found to affect the risk of having an accident (6). Nevertheless, because an effective surveillance system is not in place, data on childhood injuries in our country is rather scarce. And also, due to the fact that changes in life conditions and the environment of children will change risk factors for injuries over time, epidemiologic studies should be repeated in order to improve primary preventive measures.

There is a lack of research on parents’ attitudes to injury prevention measures as well as the parental adherence to these measures in our country. This study was planned to provide such data and it was also aimed at exploring the association between parents’ adherence and demographic factors.

## MATERIALS AND METHODS

This was a descriptive study conducted among the parents of all children younger than ten years of age admitted to Hacettepe University Children’s Hospital Outpatient Clinics for any reason and who agreed to be involved in the study. Suicide, assault, physical/sexual abuse, and cases with a history of psychiatric disorders were excluded. All the parents who agreed to participate in the study gave written and verbal consent, and the data were collected using a “face-to-face interview” technique between March 2013 and April 2014. The study was approved by the local ethics committee. The response rate was 83% and there were 30 incomplete questionnaires that were not used for analysis.

The sociodemographic characteristics, medical history of participating children and, the frequency, type, and place of unintentional injuries if they have experienced in the past composed the first part of the questionnaire. Serious injuries were considered to be any injury that requires hospital admission. The second part of the questionnaire was prepared to evaluate parents’ adherence to injury prevention rules. In order to adapt to the local sociocultural situation, this section of the questionnaire was modified by the researchers mainly based on The Injury Prevention Program (TIPP) parent questionnaire [the Framingham Safety Survey (FSS)] and the literature (8,9). In counselling the parents and children about adopting behaviours aimed at preventing injuries, we used the FSS, a systematic method for paediatricians to identify at-risk behaviour. The TIPP parent survey was found to be a reliable measure for evaluating the idea of injury prevention consciousness and attitudes (10). In our study, the second part of the questionnaire included questions (yes-no-sometimes type) about parents’ attitudes and adherence to injury prevention measures and was categorized according to the children’s age (Table 1). Firstly, a reviewed questionnaire was tested as a pilot study and required corrections were done.

A total score was calculated to indicate the adherence of the parents to the injury prevention rules by adding together the scores of each answer given in each age group. Answers for each item given by the parents were scored as wrong (0), sometimes (1) or correct (2). The score for each item was added and the result normalized to 100 points. Only complete questionnaires were used for analysis.

Data were analyzed using SPSS version 21.0 for Windows software (SPSS Inc., Chicago, IL). Results were reported as mean ± standard deviation (SD) values or n (%) where appropriate. The normality of the data distribution was checked using the Kolmogorov-Smirnov test in the whole group and the subgroups. A Student’s t-test was used to compare scores between subgroups. Differences among subgroups were studied by one-way ANOVA and post hoc comparisons were performed using the Duncan test. To identify the child-family predictors of scores we carried out to multiple linear regression analysis. A p-value of <0.05 was considered to indicate statistical significance.

## RESULTS

One thousand one hundred and twenty-six children and parents agreed to participate in the survey. We obtained answers predominantly from mothers (69.8%), whose mean age was 31 (SD: 5.8) years. The median number of children was two, and the median family size was four people. Some of the sociodemographic characteristics of the participating children are shown in Table 2, 3. Some 13.8% of children had a history of at least one serious injury. Among the children with a serious injury history, 10.2% had experienced two and more serious injuries.

Overall scores ranged from 12.5 to 95.0, with a mean of 54.6 (SD: 14.2) points. The total injury prevention scores of parents decreased significantly as their children’s age increased (p<0.001). There was no significant association between the injury prevention scores and gender of children. Although parents of children with a serious injury history had significantly lower injury prevention scores, after age group adjustment this significance disappeared (Table 2).

Parents’ educational backgrounds and ages were significantly related to their total scores (p<0.001). It was also found that parents with first child had higher scores related to injury prevention (p<0.001) (Table 3). Approximately three-quarters of the parents said that they had information about injury prevention, and their total scores were found to be significantly higher than parents without any source of information (p<0.001). The most common sources of information were the media (Internet, television or newspapers, 36.0%), first-aid courses (30.0%), friends and family members (29.7%), and doctor visits (4.3%).

Multiple linear regression analysis was used to evaluate the association between scores and child-parent parameters [children’s characteristics (age, sex, birth order, chronic illness, injury history) and family characteristics (maternal age, education, occupation and statement about knowledge of injury prevention, paternal age and education, household size, any carer other than mother)]. This analysis revealed that injury prevention scores increased significantly with high education and maternal occupation. However, scores were shown to decrease significantly with increased child age and family size (Table 4).

When we evaluated the answers of parents to the survey questions by children’s age groups, in the 0-1 year age group, parents’ adherence rates to injury prevention measures were found to be between 14.1% and 97.8% (Table 1). In this age group, 85.9% of parents did not have a working fire extinguisher in their homes. With regard to burn safety rules, approximately 86.0% of parents reported that they never drank or carried hot liquids when children were nearby. Only 51.1% of parents kept plastic bags and balloons away from their children. The prevalence of infant walker usage was found to be 28.3%. The best-answered question in this group was about water safety: only two parents said that they left their babies alone in or near a pool, bath, bucket, or toilet.

In the 1-4 years age group, parents’ adherence rates to injury prevention measures ranged from 3.7% to 95.4% (Table 1). Some 31.1% of parents were reported to use child safety furniture products, and 36.9% of them use electrical outlet safety covers. Unfortunately, only 3.7% of the parents were reported to know the poison helpline number for this age group. Adherence rates for prevention measures related to auto safety were low: only 20.7% of parents were reported to use a car safety seat on every trip. In this age group, 35.6% of children played in or near the street. The adherence rate for toy safety was high: 71.2% of parents reported that they always check their child’s toys for safety hazards.

In the 5-9 years age group, parents’ adherence rates ranged from 2.1% to 90.1% (Table 1). Only 29.3% of parents used electrical outlet safety covers. A proportion of 43.0% of parents reported that their children knew the bicycle safety rules, but only 2.1% of children wore a bicycle helmet. Although adherence rates for poisoning prevention rules were high, 19.9% of parents reported that they kept some cleaning, hazardous and chemical products in packages other than their original ones.

When we explored the power of our study, we found that group sample sizes of 156 (cases with injury) and 970 (cases with no injury) achieve 70% power to reject the null hypothesis of equal means when the population mean difference is μ1 - μ2 = 52.0 - 55.0 = -3.0 with a standard deviation for both groups of 14.0 and with a significance level (alpha) of 0.05 using a two-sided two-sample equal-variance t-test. Also, group sample sizes of 310 (mothers with no information about injury prevention) and 245 (mothers with information about injury prevention/first-aid courses) achieve 100% power to reject the null hypothesis of equal means when the population mean difference is μ1 - μ2 = 50.3 - 59.4 = -9.1 with a standard deviation for both groups of 14.1 and with a significance level (alpha) of 0.05 using a two-sided two-sample equal-variance t-test.

## DISCUSSION

Unintentional injuries affect millions of children and families each year, and many of these require hospital treatment (11,12,13,14,15). Our findings are quite similar to those in the literature. Among the children with a serious injury history, approximately one in ten experienced two or more serious injuries. Unintentional injury frequency was recently determined as 12.6%, and 10.1% of them had a two-injury history in another study from our country (6). This indicates that there is something wrong with the injury prevention strategies in Turkey so we could not prevent these children from repeated unintentional injuries.

In our study, the age of the child, parental educational level, maternal occupation, household family size, and parental statement about knowledge of injury prevention were found to be associated with parents’ injury prevention adherence. Previous studies indicate that children’s ages are related to injury prevention practices. In many studies, it was found that parents provide closer supervision for younger children (16,17). Eichelberger et al. (18) found that mothers of younger children have more correct injury prevention behaviours than parents of older children. Mothers of children aged >1 year defined fewer risks than mothers of children aged ≤1 year in a study from our country (6). In another study, it was found that parents of children younger than 2.5 years were more likely to use injury prevention rules than parents of older children (19). In our study, parents’ adherence to safety rules decreased significantly as children’s age increased. These results may be due to parents of younger children being more careful and alert to situations that might be dangerous for their children.

Parents’ educational background and mother’s occupation were also significantly associated with parents’ adherence to injury prevention measures in our study. Mother’s education level is directly proportional to injury recognition capacity, which is consistent with some previous studies (20,21). For example, it was found that children with mothers educated to primary school level had a 1.5 times greater injury risk than children with university graduate mothers (22). The total number of injury risks found by mothers was related to the educational background in another study (6). In our study, working mothers had higher scores than non-working mothers. This may be because working mothers are more sensitive to home-based risks. Another explanation for this is that working mothers’ educational levels were significantly higher than those of non-working mothers in our study (p<0.001), which may be the reason for higher scores and is consistent with some previous studies in the literature (6,20).

Although multiple linear regression analysis did not find an association between scores and birth order of the child, parents of first born child had higher total injury prevention scores in our study. Third or later-born children were found to have a 5.7 times higher risk of injury in one study (20). Another study found that mothers of first-born children spend more money on child safety devices (21). Because they have no experience, parents with the first child may depend more on parenting and injury prevention rules. As children grow they gain more experience, and the increased assurance and acceptance in their parenting skills could weaken the parents’ adherence to injury prevention rules.

It is important to know from whom parents can obtain the knowledge needed to improve their injury prevention information. Family members and the media were the most frequently mentioned sources of information about child safety in the literature (2,23). Ablewhite et al. (24) found that mothers prefer to use home safety advice from other parents instead of professionals. We found a similar result to the literature: the most frequently cited source was the media (the Internet, newspapers, and television). Social networks in particular are an important source of child safety information. Because parents find safety advice from other parents and the media more useful, suitably trained parents and social networks can be used for this purpose. Hospitals and other health services were not frequently cited sources of information. Doctor visits account for only 4.3% of them. This result shows us that not all pediatricians regularly counseled on injury prevention in our country. On the other hand, it is appropriate for paediatricians to incorporate child safety programmes into primary care. Children are regularly seen for minor illnesses, vaccinations, and child health supervision. This gives us various opportunities for interventions to promote childhood safety practices.

Parents have a critical role in reducing their children’s unintentional injury exposure risk by using safety equipment. For example, in New Zealand and the United States the majority of homes have smoke alarms (respectively, 80.5% and 96.8%) (16,25). On the other hand, a study conducted in the EU found that only 6.0% of houses have working smoke detectors (2). In our study, only 12.4% of parents mentioned that they have a fire extinguisher and approximately 17.0% of them have a working smoke alarm. Parental lack of knowledge about the causes of unintentional injuries or low parental adherence to safety rules can be a significant barrier to the safety of children. Low parental adherence to safety rules is found to be related to information deficit, parents’ negligence, and/or low income (which prevents the buying of safety products), according to the literature. Ongoing education and legislative regulations are effective in terms of injury control.

Vincenten et al. (2) reported that the most common parental practice for preventing childhood injuries was to keep hazardous products out of children’s reach. Although approximately 80.0% of parents keep medicines and household cleaners out of children’s reach in our study, 19.9% of them reported that they do not keep them in their original packages. In order to prevent this dangerous behaviour and reduce child deaths from poisoning, increased parental awareness about poisoning must be combined with legislation for child-resistant packaging.

Every year nearly 30% of all injury-related deaths are due to traffic injuries (3). Even in developed countries, the main concern for parents regarding their children was the risk of being involved in a motor vehicle accident (2,26,27,28). Child passenger safety seats (CSSs) are produced to protect children from crash-related injuries. They can decrease mortality and morbidity by up to 71% when correctly used (29). In developing countries, although most of the parent drivers had positive attitudes towards CSSs, the rate of usage was extremely low (27,28,29). Despite the fact that there is a mandatory rule in our country, child safety seat usage is extremely low. Only 22.9% of children in our study used CSSs. Parents’ false perception that their children are safe enough in the arms of an adult may be one of the reasons for not using CSSs. Cost may be a potential barrier to using CSSs in Turkey, but this was not discussed in our study. In order to increase the usage of CSSs, legislative interventions must be combined with safe driving education for parents. We also found that as the child age group increases, their CSS usage decreases significantly (p<0.001). In the 0-1 age group, the CSS usage rate was nearly twice that of the other age groups. This may be due to the increased awareness and knowledge of new parents about CSS use in recent years.

Our study had some limitations. Injury prevention attitudes and adherence to prevention measures were assessed by means of a face-to-face questionnaire and this may be prone to social desirability bias. On the other hand, the face-to-face questionnaire method remains the most practical method for assessing these parameters in large groups. Secondly, the sample was predominantly from a limited geographical area. Thirdly, selection bias was also possible such that parents whose children presented with serious injury at the emergency department were likely to report low awareness of injury risk. And lastly, our study was a cross-sectional design, and no assumption can be made about causal relationships between variables.

In conclusion, our study shows that parental adherence to child safety measures aimed at decreasing the unintentional injury risk for children is not satisfactory in Turkey. According to the literature, individual counselling for injury control is one of the most important factors in achieving prevention. In particular, parents of 5-9-year-old children, big families (more than five people), parents with less than 8 years of education, and non-working mothers should be the main target groups for intervention strategies according to our study results.

## Figures and Tables

**Table 1:**
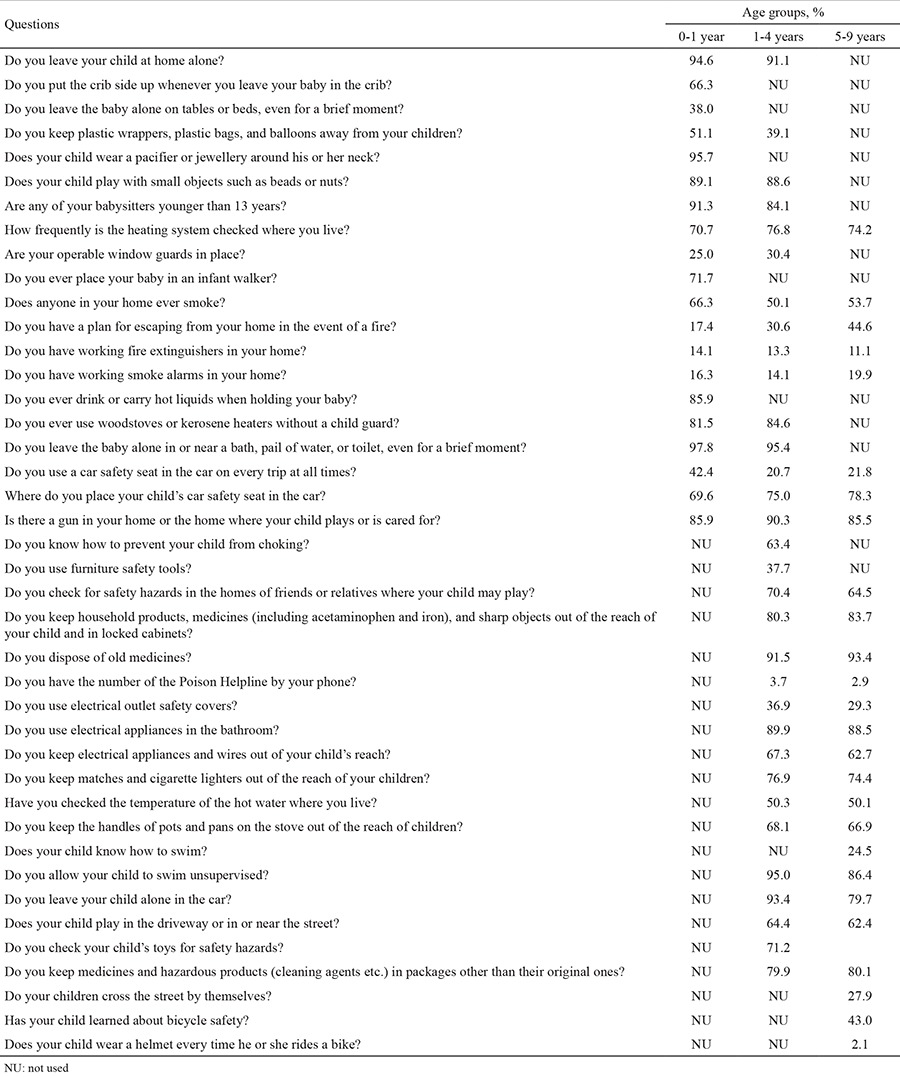
Survey questions and rates of parental adherence to injury prevention measures

**Table 2:**
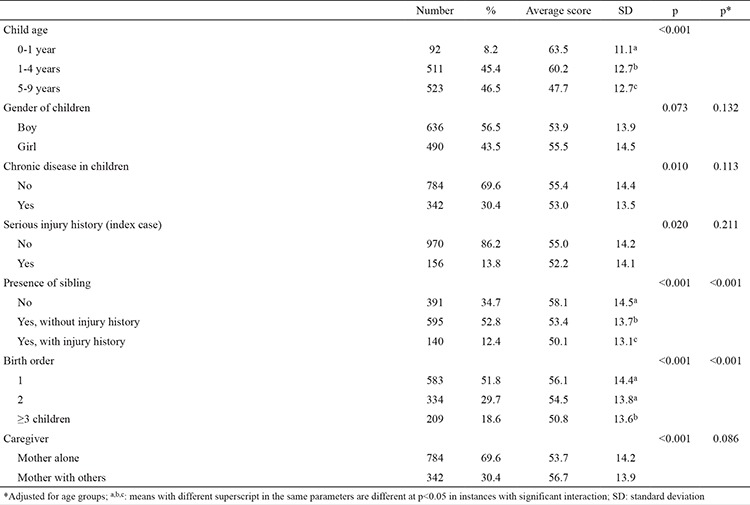
Distribution of total injury prevention scores of participating parents according to child variables

**Table 3:**
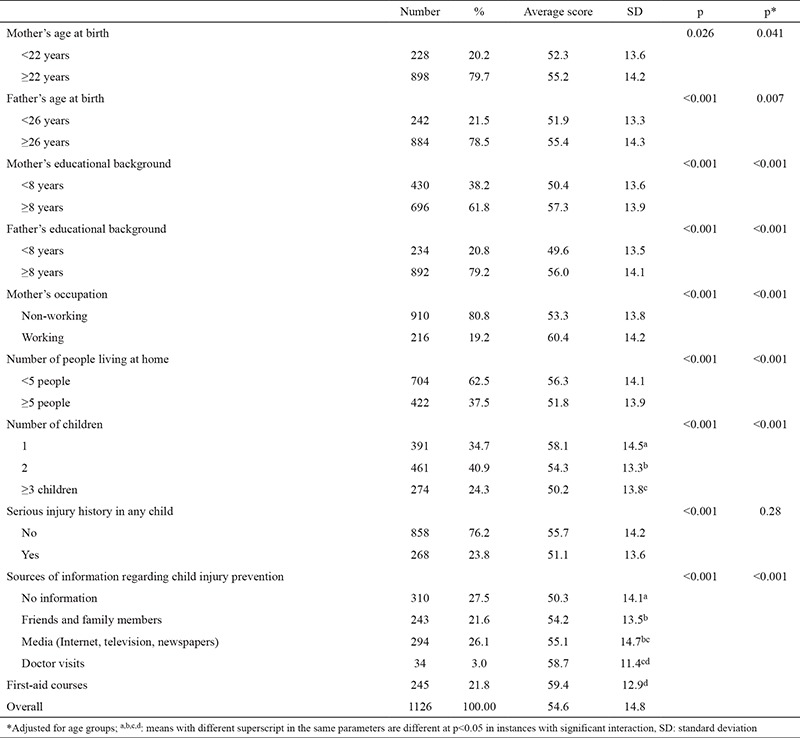
Distribution of total injury prevention scores of participating parents according to family characteristics

**Table 4:**
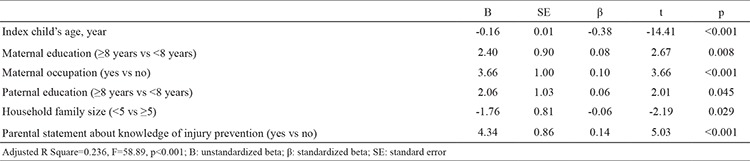
The association between scores and infant-parent parameters. Multiple linear regression analysis (stepwise)
